# Medical Progress

**Published:** 1882-11-20

**Authors:** 


### *MEDICAL PROGRESS.*

There are those engaged in the practice of medicine who discard the fact of the great progress which has, of late years, been made in medical science. They are old fogy-routine practitioners who do not read the Medical Journals, and as a consequence don't know what is going on. They reject, or affect to despise, all new drugs and new agencies, because they do not know how to use them. They are too lazy to read the Journals and improve themselves, or hold their purse-strings too tightly to devote a small pittance—perhaps the proceeds of a single medical visit—to pay for a Journal which would keep them posted on the advances of the profession. Many of this class of men are located at points where they are the only dependence, occupying perhaps good fields, and crowding out better men. The people, in their ignorance, know no better than to sustain and patronize them, and are thus placed at a most serious disadvantage.

How these careless men can reconcile it to their consciences to practice medicine in ignorance of so many valuable agencies that they could and ought to learn, we cannot understand. Among these valuable means are included the use of anæsthetics, the clinical thermometer, the hypodermic syringe, the use of veratrum, gelsemium, aconite, the bromides and many other valuable remedies and appliances, which of late years have so enlarged and strengthened the armamentarium of the practitioner.

As journalists we have a good opportunity to know this fact. It is vain that we appeal to them through circulars or otherwise, and the astonishing fact remains that scarcely one in ten of those engaged in the practice ever buy a new book or read the medical journals.

It is clearly the duty of the progressive medical men—those who read and those who write, those who feel some degree of pride in their profession—to use their influence to advance and elevate the profession by inducing practitioners everywhere to read and sustain the medical literature of the country, and to inform themselves in the advances of our noble art. And, in doing this, let not the extremely cheap and trashy Journals be sustained to the neglect of the more useful and substantial Journals in your own section. Let medical societies be organized in every neighborhood; let the brotherhood of the profession be encouraged by mingling together, thus cultivating social relations and uniting heart and hand in maintaining and advancing the great interests of the profession.

